# Interleukin-17A in Egyptian leprosy patients: a clinical, genetic, and biochemical study^☆^

**DOI:** 10.1016/j.abd.2021.09.016

**Published:** 2022-09-21

**Authors:** Azza Gaber Antar Farag, Azza Zagloul Labeeb, Amany Nagy Abdalla Gerges, Mustafa Elsayed Elshaib

**Affiliations:** a Dermatology, Andrology and STDs Department, Faculty of Medicine, Menoufia University, Egypt; b Microbiology and Immunology Department, Faculty of Medicine, Menoufia University, Egypt; c Leprosy and Dermatology Clinic, Ministry of Health, Shebin Elkoom Teaching Hospital, Egypt; d Medical Student, Faculty of Medicine, Menoufia University, Egypt

**Keywords:** Interleukin-17A, Leprosy, Polymorphism, single nucleotide

## Abstract

**Background:**

Leprosy represents a long-term communicable disease resulting from *Mycobacterium leprae* infection. IL-17A is one of the pro-inflammatory cytokines that protects humans against many fungal and bacterial pathogens.

**Objective:**

To investigate IL-17A (rs2275913) gene polymorphism and its circulating level in leprosy patients, and to correlate the detected results with different clinical aspects of leprosy in the investigated patients.

**Methods:**

60 patients with leprosy, and 29 age and sex-matched volunteers were investigated for IL-17A serum level and IL-17A single nucleotide polymorphism (SNP) by ELISA and RFLP-PCR respectively.

**Results:**

IL-17A serum level was significantly higher in leprosy patients than in controls (p = 0.034), and in TL than LL (p = 0.017). IL-17A (rs2275913 A/G) G allele and GG genotype were associated significantly with LL (p = 0.005and 0.001 respectively). IL-17A (rs2275913 A/G) AG genotype carriers demonstrated the highest IL-17A serum levels; however, its lowest levels were found in IL-17A (rs2275913 A/G) AA genotype carriers (p = 0.005). Grade 2 disability (p = 0.030) and positive slit skin smear (SSS) (p = 0.005) were significantly associated with IL-17A (rs2275913 A/G) GG genotype.

**Study limitations:**

The small number of studied subjects.

**Conclusions:**

IL -17A may have a pivotal role in leprosy pathogenesis. IL-17A (rs2275913) GG genotype plus G allele might be related to the development of LL in the Egyptian population.

## Introduction

Leprosy is considered a long-term infectious disease caused by *M. leprae*. It can affect mostly nerves, skin, respiratory tract, and eyes causing their damage. The symptoms of leprosy may appear within a year, but for some people, symptoms may take 20 years or more to occur.^1^

Leprosy remains a major health problem in some countries^2^ including Egypt which is still one of the 22 countries of global priority.^3^

Leprosy is classified into two polar types; tuberculoid Leprosy (TL) and lepromatous Leprosy (LL), plus three types in between; borderline Tuberculoid (BT), borderline Borderline (BB), and borderline Lepromatous (BL). TL is characterized by the presence of a few bacilli, paucibacillary (PB), and vigorous cell mediate immunity. However, in LL, numerous bacilli, multibacillary (MB), and inefficient cell mediate immunity were found.^4^

In TL, there are a high proportion of CD4+ T-cells with increasing type 1 cytokines. On the other hand, LL shows a predominance of CD8+ T-cells plus type 2 cytokines. The aforementioned data demonstrate the immunopathological interactions between type 2 and type 1 cytokine and activated macrophage products (monokines) in leprosy.^5^

However, a certain T-cell subset was shown to produce cytokines that could not be classified conferring to the Th1-Th2 scheme. Interleukin (IL)-17 was among these cytokines and T-cells producing IL-17 were named Th17 cells.^6^

IL-17A is a protein that in humans is encoded by the IL-17A gene. It is a pro-inflammatory cytokine.^7^ Originally IL-17A was supposed to be exclusively produced by T-cells, but it is also secreted by many cells including dendritic Cells (DC), macrophages, natural Killer (NK), and γδ-T cells.^8^

IL-17A acts on structural cells such as epithelial cells, fibroblasts, and keratinocytes in various tissues including skin. These cells express IL-17 receptors and produce many cytokines required for inflammation such as granulocyte Colony-Stimulating Factor (GCSF) and IL-6 as well as chemokines to attract neutrophils and macrophages to the inflamed tissues. These inflammatory cells (neutrophils and macrophages) can both clear the infection and initiate pathogenic inflammation.^6^

Additionally, the binding of IL-17A to IL-17 receptor causes important conformational changes that enable the binding of signaling adaptors, such as TRAFs proteins. The binding of these signaling adaptors triggers activation of several signaling pathways, including nuclear Factor Kappa B (NF-κB), pathways of mitogen-activated protein kinase.^9^

Activation of these pathways leads to the production of many inflammatory cytokines like IL-1β and tumor Necrosis Factor-Alpha (TNF-α) and chemokines, which attract macrophages as well as antimicrobial products such as mucins and defensins. Through its inflammatory effects, these products help in the elimination of many foreign pathogens.^10^

It was demonstrated that IL-17A protects the human body against several bacterial and fungal infections caused by invading pathogens.^9^

The aim of the present study was to shed light on the hypothesized role of interleukin-17A in leprosy pathogenesis, through analysis of IL-17A (rs2275913) gene polymorphism plus its serum levels in leprosy patients, and to correlate the detected results with different clinical aspects of leprosy in the investigated patients.

## Materials and methods

The current study was a case-control study. It was conducted on 60 patients having various types of leprosy and 29 age and sex-matched healthy volunteers with no past or family history of leprosy (control group). Leprosy patients and control group were collected from Dermatology and Leprosy Clinic, Menoufia Teaching, and university Hospitals. The laboratory part of the study was done at the Microbiology and Immunology Department, Faculty of Medicine, Menoufia University.

This study was approved by the Ethical Committee of Human Rights in Research at Menoufia University. Informed written consent was signed by each participant after informing them about the study.

Patients from both sexes that had been confirmed as leprosy by clinical aspects of their lesions, previous positive SSS, and/ or histopathology evaluation were included.

Subject with any of the following conditions was excluded from this study: 1- systemic diseases (e.g., diabetes mellitus, cirrhosis, systemic infection, renal failure, collagen disease, or malignancy); 2- endocrinopathies (such as Cushing’s syndrome, and hyper-and hypothyroidism); 3- anti-inflammatory drugs or hormonal medicines, such as steroids. 4- pregnancy and lactation.

A sheet was performed for all patients, including name, age, gender, residence, occupation, and history of leprosy state. To assess socioeconomic Level (SEL), the authors used a scale including 7 domains (education and cultural domain, occupation domain, family possessions domain, family domain, home sanitation domain, economic domain, and health care domain). Each participant was asked about occupations, an account income, and educational levels. SEL total score is 84. A high SEL was indicated as at least 70% (59–84), a medium level as 40% to less than 70% (34–58), and a low level as less than 40% (less than 34).^11^

The dermatological examination was done including a complete clinical assessment of skin lesions to determine the distribution, number, and consequently its clinical variants. In addition, a neurologic examination of possible affected nerves was performed. The studied leprosy patients have categorized in relation to the Madrid (1953) criteria to tuberculoid leprosy, borderline leprosy, and lepromatous leprosy.^12^

Under the complete aseptic condition, 5 mL of blood was withdrawn from every subject and divided into two parts. The first part (2 mL) was kept in a plain tube, left at 37 °C for 30 min to clot, and after that centrifuged at 4000 r.p.m for 10 min. The serum obtained was put into aliquots and kept in −80 °C till the time of analysis for determination of IL-17A serum level by ELISA kits (Neo Bioscience Technology Co., Ltd, Shenzhen, People’s Republic of China) according to the manufacturer’s instructions.

The second part (3 mL) was transferred into an EDTA vacutainer tube immediately and stored for DNA extraction. DNA extraction was done using Gene JET™ Whole Blood Genomic DNA Purification Mini Kit (THERMO SCIENTIFIC, EU/Lithuania), following the manufacturer's instructions. SNP to IL17A -197G\A (rs2275913) genotyping was achieved by Polymerase Chain Reaction-Restriction Fragment Length Polymorphism (PCR-RFLP). Primers sequences for IL17A -197G\A were 5′-AACAAGTAAGAATGAAAAGAGGACATGGT-3′(sense) and 5′- CCCCCAATGAGGTCATAGAAGAATC-3′(anti-sense).

Amplification of PCR was done in a volume of 30 μL mixture containing 100 ng genomic DNA, 1.0 μM of each primer, 200 μM of each dNTP, 2.0 mM of MgCl_2_, 3 μL 10 × PCR buffer, and 1.5 *U* Taq DNA polymerase (Invitrogen Life Technologies, Grand Island, NY, USA). Restriction enzymes XagI (THERMO SCIENTIFIC, EU/Lithuania) were used to distinguish IL17A -197G\A (rs2275913) (102,68, 34 bp) genotypes. PCR product and enzyme were well mixed and incubated at 37 °C for 1.5 h. The pattern of the digestion products migration on 3% agarose gel allowed the identification of the IL17A -197G\A genotypes. Genotyping was performed blindly and most equally in number of cases and controls. G genotype (68 + 34 bp), A genotype (102 bp), and AG genotype (102, 68, 34 bp) were identified.^13^

### Statistical analysis

The data collected were analyzed statistically by SPSS (version 20) on IBM compatible computer. Two types of statistics were done: 1) Descriptive statistics: which were expressed as mean plus standard deviation (X ± SD) as well as median and range for quantitative data or number and percentage (No and %) for qualitative data; 2) Analytic statistics: including Chi-Square (χ^2^), Mann-Whitney, Kruskal-Wallis, and *Z* tests were used; p-value ≤ 0.05 was statistically significant.

## Results

### Socio-demographic and clinical data

The age of the selected cases ranged from 19 to 82 years. They were 44 (73.3%) males and 16 (26.7%) females, and 83% of the studied patients were from a rural area. Fifteen (25%) leprosy patients had TL, 25 (41.7%) patients were LL and 20 (33.3%) cases were BL patients.

Compared to controls, most leprosy patients (90%) were significantly low SEL (p = 0.001) and 13.3% of them had a positive family history of leprosy (p = 0.039) (Table 1).

**Table 1:** Socio-demographic and clinical data of studied subjects.

Socio-demographic characteristics	Patients	Controls	Test of significance	p-value
	(n = 60)	(n = 29)		
Age (years)				
Mean ± SD	51.97 ± 13.67	45.92 ± 14.16	*U* test = 1.93	0.056
Median	52	51		
Range	19‒82	16‒78		
Gender	n (%)	n (%)	χ^2^	
Males	44 (73.3)	23 (79.3)	0.38	0.540
Females	16 (26.7)	6 (20.7)		
Residence				
Rural	50 (83.3)	22 (75.9)	0.71	0.401
Urban	10 (16.7)	7 (24.1)		
Occupation				
Working	47 (78.3)	18 (62.1)	2.63	0.105
Not working^a^	13 (21.7)	11 (37.9)		
Socio-economic level				
High	1 (1.7)	8 (27.5)	30.89	<0.001^b^
Middle	5 (8.3)	11 (37.9)		
Low	54 (90.0)	10 (34.5)		
Family history for leprosy				
Positive	8 (13.3)	0	4.25	0.039^b^
Negative	52 (86.7)	29 (100.0)		
Type of leprosy				
TL	15 (25)	‒	‒	‒
BL	20 (33.3)			
LL	25 (41.7)			
Degree of disability				
Grade 0	15 (41.7)	‒	‒	‒
Grade 1	10 (16.7)			
Grade 2	25 (41.7)			
Slit skin smear		‒	‒	‒
Positive	35 (58.3)			
Negative	25 (41.7)			
Disease duration (years)				
Mean ± SD	7.10 ± 5.14	‒	‒	‒
Median	6			
Range	2 months‒25			

χ^2^, Quisquare test; n, number; *U*, Mann-Whitney test; TL, Tuberculoid leprosy; LL, Lepromatous leprosy; BL, Borderline leprosy.

a Not working included students and retired.

b Significant.

### IL-17A serum levels

There was significant higher IL-17A serum levels in leprosy patients (4.84 ± 6.79) than the control group (2.04 ± 2.25) (p = 0.034) (Table 2), which was significantly higher in TL (4.52 ± 5.87) than LL (2.91 ± 1.69) (p = 0.017) (Table 3).

**Table 2:** IL-17A serum levels and gene polymorphism in leprosy patients and control group.

IL-17A	Patients	Controls	test	p-value
	(n = 60)	(n = 29)		
Serum IL-17A (pg/mL)				
Mean ± SD	4.84 ± 6.79	2.04 ± 2.25	*U* = 2.03	0.034^a^
Median	2.68	2.44		
Range	1.5‒30.03	1.41‒22.44		
IL-17A (rs2275913) SNP	n (%)	n (%)	χ	
Genotypes				
GG	35 (58.3)	16 (55.2)	0.22	0.898
AG	15 (25.0)	7 (24.1)		
AA	10 (16.7)	6 (20.7)		
Alleles	(n = 120)	(n = 58)		
G	85 (70.8)	39 (67.2)	0.24	0.625
A	35 (29.2)	19 (32.8)		

SD, Standard Deviation; pg/mL, Picogram per millilitre; χ^2^, Quisquare test; *U*, Mann-Whitney test.

a Significant.

**Table 3:** IL-17A serum levels and gene polymorphism in different types of leprosy patients.

IL-17A	Patients (n = 60)			p^a^	P1^b^	P2^b^	P3^b^
	TL	BL	LL				
	(n = 15)	(n = 20)	(n = 25)				
Serum IL-17A(pg/mL)							
Mean ± SD	4.52 ± 5.87	7.48 ± 10.10	2.91 ± 1.69	0.059	0.230	0.017^c^	0.253
Median	3.10	2.65	2.54				
Range	1.52‒25.6	1.74‒30.03	1.59‒10.63				
IL-17A (rs2275913) SNP	n (%)	n (%)	n (%)		χ^2^	χ^2^	χ^2^
Genotypes							
GG	6 (40.0)	10 (50.0)	19 (76.0)	0.001^c^	0.009^c^	0.071	0.011^c^
AG	8 (53.3)	2 (10.0)	5 (20.0)				
AA	1 (6.7)	8 (40.0)	1 (4.0)				
Alleles	(n = 30)	(n = 40)	(n = 50)				
G	20 (66.7)	22 (55.0)	43 (86.0)	0.005^c^	0.324	0.041^c^	0.001^c^
A	10 (33.3)	18 (45.0)	7 (14.0)				

P1, TL versus BL; P2, TL versus LL; P3, LL versus BL; χ^2^, Quisquare test.

a Krukall-Wallis test was used.

b Mann-Whitney test was used.

c Significant.

### Relationship between serum IL-17A levels and clinical data in leprosy patients

**Figure 1:**
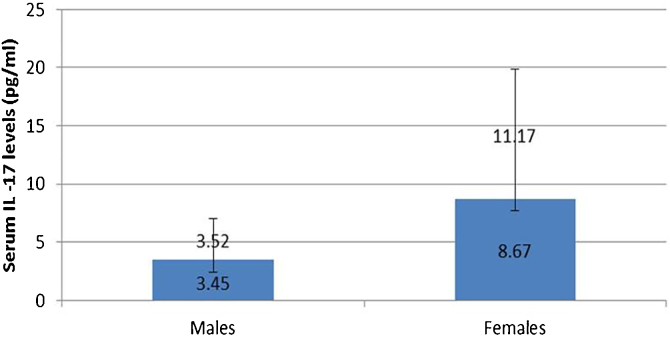
Serum IL-17A levels for males and females in leprosy patients.

IL-17A serum level was not affected by any personal or clinical data of the studied leprosy cases except for gender as a significantly higher serum IL-17A levels were reported in female than male leprosy patients (p = 0.007) (Fig. 1).

### IL-17 (rs2275913A/G) genotypes and alleles

**Figure 2:**
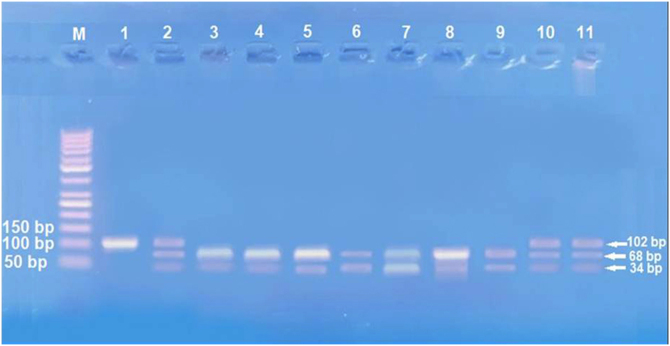
Agarose gel electrophoresis images for IL-17A genotypes, GG genotype 3, 4, 5, 6, 7 and 9 lanes, AG genotype 2, 10 and 11 lanes, AA genotype 1 and 8 lanes.

Studying IL-17A (rs2275913A/G) SNP (Fig. 2) showed that there was a statistically non-significant difference between leprosy patients and the control group regarding IL-17A (rs2275913A/G) genotypes (p = 0.898) and alleles distribution (p = 0.625) (Table 2). However, significant differences between the three types of leprosy patients regarding percent distributions of IL-17A genotypes (p = 0.001) and alleles (p = 0.005) were demonstrated, as IL-17A (rs2275913A/G) GG genotype besides G allele were associated significantly with LL, while AG genotype was significantly associated with TL cases (Table 3).

### Relationship between IL-17A genotypes and clinical data in leprosy patients

**Figure 3:**
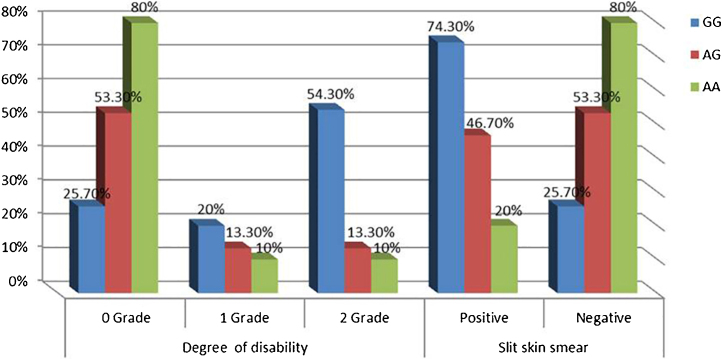
IL-17A genotypes regarding disability grades and SSS in leprosy patients.

Non-significant differences were found among IL-17A (rs2275913A/G) genotypes regarding personal and clinical data of leprosy patients except grade of disability and SSS as a higher percentage of grade 2 disability and positive SSS were associated significantly with IL-17A (rs2275913A/G) GG genotype than AG and GG genotypes (p = 0.030 and 0.005; respectively) (Fig. 3).

### Relationship between IL-17A serum levels and IL-17A (rs2275913A/G) genotypes

In leprosy patients, there was a statically significant relationship between IL-17A (rs2275913A/G) genotypes and serum IL-17A levels as the highest levels were found in IL-17A (rs2275913A/G) AG genotype carriers and the lowest levels were found in AA genotype carriers (p = 0.005). However, in the control group, a non-significant relationship was found (p = 0.505) (Table 4).

**Table 4:** Relationship between IL-17A serum levels and IL-17A (rs2275913) genotypes in leprosy patients and control group.

IL-17A (rs2275913) genotypes	Serum IL-17A levels (pg/mL)	
	Patients	Controls
	(n = 60)	(n = 29)
GG		
Mean ± SD	4.95 ± 7.28	4.65 ± 5.56
Median	2.6	2.48
Range	1.52‒30.03	1.71‒22.44
AG		
Mean ± SD	6.08 ± 7.76	2.39 ± 0.65
Median	3.11	2.20
Range	1.59‒25.60	1.64‒3.54
AA		
Mean ± SD	2.59 ± 0.57	2.42 ± 0.77
Median	2.55	2.52
Range	1.74‒3.94	1.41‒3.64
Kruskall-Wallis test	10.69	1.37
p-value	0.005^a^	0.505

SD, Standard Deviation; pg/mL, Picogram per milliliter.

a Significant.

## Discussion

In various pathogenic conditions, IL-17A can clear the infection and initiate an inflammatory reaction that could be mediated through the NF-κB pathway,^9^ production of many inflammatory cytokines such as TNF-α, IL-1β,^10^ G-CSF, and IL-6 as well as chemotaxis of macrophages and neutrophils to the inflamed tissues.^6^

In agreement with these data, the authors demonstrated that the circulating IL-17A levels were of significantly higher mean values in leprosy patients than controls and in TL than LL. This high level may represent the body's defense mechanism against *leprae bacilli* infection.

In line with the present result, Sakdiah et al.^14^ studied 40 Indonesian patients with leprosy versus 40 controls. They showed that IL-17 serum levels in leprosy patients were higher than in controls. Also, Santos et al.^15^ investigated 51 Brazilian leprosy patients and 23 controls. The authors revealed that IL‐17A concentrations were elevated in TL patients than in LL and controls. Additionally, they demonstrated more CD4+ IL-17A + cells in skin lesions from TL than those from LL patients. Santos and his co-workers reported a significant association between Th17 cells with the effective inflammatory response present in the PB forms.^15^

In this study, the authors found a significantly high serum level of IL-17A in female than male patients. Tsuboi et al.^16^ studied 409 patients (200 males, 208 females) in Japan and found elevated IL-17A serum levels among females having depression. Therefore, the authors could speculate that the increased IL-17A serum levels in female patients may possibly be explained by that these females could be suffering from psychiatric disorders including depression resulting from their disease state. Moreover, females are more susceptible to developing many inflammatory and autoimmune disorders than males, and increased IL-17 serum levels may participate in these disorders even before appearing in their clinical picture.

Although *M. leprae* can infect many people, few become sick, which can be attributed, in part, to the influence of genetic inheritance on the immune response. Many SNPs are taught to be linked with the production of cytokines and chemokines have been implicated with clinical forms of leprosy and reactional episodes.^17^, ^18^

In the existing study, the authors found a statistically non-significant difference between leprosy patients and the control group regarding IL-17 (rs2275913A/G) genotypes and allele distribution. However, a comparison between leprosy subgroups (LL, TL, and BL) revealed that IL-17 (rs2275913A/G) GG genotype in addition to the G allele was associated significantly with LL.

In line with the present result, Aquino et al.^19^ studied 150 Brazilian patients with leprosy and 150 household contacts. They found that genotype frequencies had non-significant differences in the overall leprosy patients and controls.^19^ Also, Escamilla-Tilch et al.^20^ investigated 75 Mexican Mestizo leprosy patients versus 69 controls. Sixty-two patients were classified as LL, 2 as TL, 2 as BL, and 9 as dimorphic. They found no association between IL-17A (rs2275913A/G) gene SNPs and leprosy.^20^ Therefore, the present study confirmed the evidence of a lack of relationship between IL-17A (rs2275913A/G) gene polymorphism and leprosy susceptibility.

Regarding leprosy subgroups, although the authors reported a significant link between IL-17A (rs2275913A/G) G allele and GG genotype with LL, Escamilla-Tilch et al.^20^ did not find this association. This difference could be explained on the bases of environmental and socioeconomic factors that may influence the development of different pathologies. Moreover, Escamilla-Tilch and his collages investigated only 4 cases (2 as TL and 2 as BL) versus 62 having LL, and this sample size may be not suitable for statistical assessment.^20^

In this study, the authors noticed that a higher percentage of grade 2 disability and positive SSS were associated significantly with IL-17A (rs2275913A/G) GG genotype confirming the current demonstrated a higher predominance of GG genotype in the study’s LL patients.

Among the studied IL-17(rs2275913A/G) genotypes, AG genotype carriers demonstrated the highest IL-17 serum levels. This could be clarified by the predominance of AG genotype in the current studied TL cases, which is characterized by good immunity and high cell mediate the immune response.

However, Aquino et al.^19^ showed that IL-17A (rs2275913) AA genotype was related to high levels of IL-17A in the serum. This difference could be attributed to racial and genetic factors. Aquino et al.^19^ studied a mixed population from southern Brazil, however, the authors investigated the Egyptian population who belong to the African race. According to Pedia for IL-17A (rs2275913) snipe, IL-17A (rs2275913) A allele was found only in 1.8%–21.1% in African peoples. In agreement with Pedia snipe data, the authors found IL-17A (rs2275913) A allele was lower than G allele (29.2% and 70.8% respectively), and AA genotype was the lowest genotype (10/60, 16.7%) in leprosy patient group. Recently, Mazurek-Mochol et al.^21^ reported that the A allele was associated with increased production of IL-17. Therefore, decreased IL-17A (rs2275913) A allele and AA genotype in the present studied cases could also explain that difference.

## Conclusions

IL-17A may have an active role in leprosy pathogenesis. Although IL-17A (rs2275913A/G) polymorphisms have a non-significant role in the genetic susceptibility to the development of leprosy, IL-17A (rs2275913A/G) GG genotype, as well as G allele, were of significant value in and linked to the progression to LL in the Egyptian population.

## Financial support

None declared.

## Author’s contributions

All authors should have made substantial contributions to all of the following:

Azza Gaber Antar Farag: Critical literature review; Study conception and planning.

Azza Zagloul Labeeb: Data collection, analysis, and interpretation.

Amany Nagy Abdalla Gerges: Data collection.

Mustafa Elsayed Elshaib: Statistical analysis.

## Conflict of interest

None declared.
